# Time to First-Line Antiretroviral Treatment Failure and Its Predictors among HIV-Positive Children in Shashemene Town Health Facilities, Oromia Region, Ethiopia, 2019

**DOI:** 10.1155/2021/8868479

**Published:** 2021-08-17

**Authors:** Endale Zenebe, Assefa Washo, Abreham Addis Gesese

**Affiliations:** ^1^Jimma University, College of Public Health and Medical Science, Department of Epidemiology, Jimma, Ethiopia; ^2^Hawassa College of Health Science, Department of Public Health, Hawassa, Ethiopia

## Abstract

With expanding pediatric antiretroviral therapy access, children will begin to experience treatment failure and require second-line therapy. In resource-limited settings, treatment failure is often diagnosed based on the clinical or immunological criteria which occur way after the occurrence of virological failure. Previous limited studies have evaluated immunological and clinical failure without considering virological failure in Ethiopia. The aim of this study was to investigate time to first-line antiretroviral treatment failure and its predictors in Shashamene town health facilities with a focus on virological criteria. *Methods*. A retrospective cohort study was conducted in three health facilities of Shashamene town, Oromia Regional State, from March 1 to 26, 2019. Children aged less than 15 years living with HIV/AIDS that were enrolled on ART between January 1, 2011, and December 30, 2015, in Shashamene town health facilities were the study population. Data were extracted using a checklist, entered into EpiData version 3.1, and exported to SPSS version 20 for data analysis. Cox proportional hazard regression was used to determine the predictors of time to first-line treatment failure. *Result*. The median survival time to virological failure was 30 months with IQR of 24.42 to 44.25. Baseline WHO stages 3 and 4 with AHR = 5.69 (95% CI: 2.07–15.66) and NVP-based NNRT at initial treatment with AHR = 2.72 (1.13–6.54) were the independent predictors of time to treatment failure. *Conclusion*. The median survival time of first-line antiretroviral treatment failure was moderate in the study area as compared to other studies. The incidence density of treatment failure in this study was low as compared to other studies. The finding also demonstrated that children treated with nevirapine-based nonnucleoside reverse transcriptase inhibitors at initial and advanced WHO clinical stages at baseline were at higher risk of treatment failure.

## 1. Introduction

Worldwide, AIDS now accounts for 3% of deaths in children under five years of age—6% of those in sub-Saharan Africa, where AIDS has become one of the major killers of young children. Without HIV care, including antiretroviral therapy, the progression of HIV infection in children is particularly aggressive [1]. Sub-Saharan Africa has the largest burden of pediatric HIV in the world. A global target has been set for the eradication of pediatric HIV by 2015, but there are still so many complex issues faced by HIV-infected and affected children in the subcontinent [2].

The burden of morbidity and mortality associated with HIV infection has decreased over the past decade as access to ART has increased [3]. After more than ten years of use, highly active antiretroviral therapy (HAART) treatment's effect has been documented in all WHO European region countries reporting increased survival, decreased HIV-associated mortality, and vastly improved quality of life [4].

Delays detecting treatment failure and switching to second-line combination antiretroviral therapy (cART) are often observed in human immunodeficiency virus- (HIV-) infected children in low-middle-income countries (LMIC). Reported regimen switching rates have been lower than expected. The absence of virological monitoring is associated with delayed switching and consequent accumulation of resistance mutations to nucleoside reverse transcriptase inhibitors (NRTIs) [5, 6].

Studies in LMIC have reported high rates of virological suppression in children up to 5–6 years after treatment initiation. However, treatment failure rates of 10–34% were observed among children after 2–3 years of cART [6]. From the study done in Uganda and Mozambique, the median time to treatment failure was 379 days (IQR: 229–649) [6].

In Ethiopia, based on the 2014 estimate, 178,500 children under 15 years need ART. In 2016, there were an estimated 748,933 people living with HIV including 78,834 children, and the total estimated new HIV infections were 21,565 and among them children under 15 were 2,212. In 2015, of the total number of people who needs ART, i.e., 636,556 people, 391,844 (61.6%) accessed antiretroviral treatments; however, the number of children under 15 was only 22,967 (27.3%) [7].

The Ethiopian Pediatric HIV Cohort (EPHIC) was established to identify clinical and laboratory predictors of virological treatment failure to ultimately develop a clinical-immunological prediction rule with area under the curve of >0.80 for detecting first-line antiretroviral therapy failure (ARTF). It will also assess the performance of the current WHO guidelines for the detection of first-line ARTF in children. Many studies have reported the success of highly active antiretroviral therapy in improving clinical and immunological outcomes of children. However, as the use of HAART increases, the issue of drug resistance and subsequent treatment failure presenting as one or more of clinical, immunological, or virological ART failure appears as a challenge [8, 9].

Antiretroviral drugs are medications to be taken for life and they need follow-up to make sure they are still helping to achieve maximal viral suppression so that in the event they failed to do so, the regimen could be changed immediately [9]. Optimization of the clinical management of PLWH and prompt diagnosis of treatment failure are becoming increasingly critical in the context of lifelong treatment and limited drug availability [6].

The diagnosis of treatment failure is guided by viral load testing in high-income countries; however, this is not the case in lowest income countries. This is because of the sophisticated and expensive laboratory facility and training of personnel needed for determining viral load. As a result, in resource-limited settings, treatment failure is often diagnosed based on the clinical or immunological criteria which occur way after the occurrence of virological failure. Keeping patients on a failing regimen leads to the reversal of clinical conditions of patients to the pretreatment state and development of drug resistant strains. Once drug-resistant virus starts transmitting in the population, the consequences will be devastating. ART failure is not a common diagnosis in most centers in Ethiopia. Very few patients among the needy started on second-line ART regimens [10, 11]. Previous limited studies have evaluated immunological and clinical failure without considering virological failure in Ethiopia. This study, therefore, aims to determine time to first-line treatment failure and its predictors in Shashamene town health facilities with a focus on virological criteria, which is a gold standard for monitoring ART.

Knowing predictors that can help to predict treatment failure will help to identify those clients that are at a higher risk of failure. Armed with this information, clinicians could give such patients special attention during their follow-up and the limited resources available for diagnosing treatment failure can be used for them.

## 2. Methods and Materials

### 2.1. Study Area and Period

The study was conducted in Shashamene town, Oromia Regional State. Shashamene is a capital town of West Arsi Zone, Oromia Region, Ethiopia. The town lies on the Trans-African Highway 4 Cairo-Cape Town, about 150 miles (240 km) from the capital of Addis Ababa. The town has a total population of 264,780 in 2017, of which 133566 (50.4%) are male, 131,214 (49.6%) are female, and 43,424 are children under 15 years of age. 484 children were enrolled on ART from January 1, 2011, to December 30, 2015. The town has 8 kebeles with two public hospitals and four public health centers. Of these health facilities, one health center and two hospitals were provided ART service for the last seven years. The study was conducted from March 1 to 26, 2019.

### 2.2. Study Design

Facility-based retrospective cohort study was conducted.

#### 2.2.1. Population

All children living with HIV/AIDS, aged less than 15 years, and on ART at Shashamene public health facilities were the source population while HIV-positive children that were enrolled on ART between January 1, 2011, and December 30, 2015, are the study population. Children transferred into ART with baseline information were included in the study and children whose intake form was not completed (data that have no commencement date of ART initiation and patient age) were excluded from the study.

### 2.3. Sample Size Determination

The total number of cases from January 1, 2011, to December 30, 2015, from three facilities was 484. The power of the study was calculated by using STATA version 12. The exposure status for PMTCT and using nevirapine group regime was considered and the most significant predictor of first-line antiretroviral treatment failure, nevirapine group regime, was used, which was taken from the study conducted on predictors of virological failure in South Africa; the probability of event (probability of virological failure) in this study is 0.193, *α* = 0.05, and AHR for nevirapine group regime is 1.8 [12]. Accordingly, the calculated power of the study was 81.1%.

### 2.4. Study Variables

#### 2.4.1. Dependent Variables

The outcome variable was survival time or time to first-line treatment failure of HIV-infected children after starting HAART. The survival time was measured using months from the time of HAART initiation until the time to first-line treatment failure. Survival times of children who were still alive as of February 30, 2018, on first-line treatment, lost to follow-up, and dead were considered censored times.

#### 2.4.2. Independent Variables

Independent variables are given as follows:

  Sociodemographic characteristics: age, sex, child's primary caregiver, and nutritional history
  Baseline clinical factors: WHO clinical stages, nutritional status, opportunistic illness (OI), and TB infection
  Baseline immunological information: immunization status, CD4 count, and hemoglobin level
  Treatment-related factors: the last drug adherence, type of initial regimens, prophylaxis use, and maternal exposure status to PMTCT

### 2.5. Variable Definition

The definitions of variables are given as follows:

  Baseline measurements were those taken closest to ART initiation and within 6 months (CD4 and viral load) or 2 weeks (weight) prior to 1 week after the commencement of ART.
  CD4 count categorized as per the WHO is the appropriate classification to describe their immunological level. Children under age 1 and who had CD4 cell count <1500 cells/mm^3^; children aged between 1 and 3 years and who had CD4 cell count <750 cells/mm^3^; children aged between 3 and 5 years and who had CD4 cell count <350 cells/mm^3^; and children aged between 5 and 15 years and had CD4 cell count <200 cells/mm^3^ will be categorized as having CD4 cell count below threshold [13].
  Hemoglobin level was retrieved from record to assess anemia; for age 6–59 months, <11 g/dl; for 5 to 12 years, <11.5 g/dl; and for 12 to 15 years, <12 g/dl was categorized as anemic otherwise not anemic [14].
  Adherence to HAART was measured by the last adherence level recorded on the follow-up form and classified as good >95%, fair 85–94%, and poor <85% based on the percentage of drug dosage calculated from the total monthly doses of HAART drugs.
  Nutritional status was categorized as undernutrition for under-five children if the weight for age of a child is below (−2SD) the standard deviation score, the standard WHO weight for age z-scores. Body mass index (BMI) was calculated for children 5 years old or above, and BMI less than 16 kg/m^2^ was categorized as undernutrition [1].
  Clinical failure is a new or recurrent clinical event indicating advanced or severe immune defiance (WHO clinical stage 3 and 4 clinical condition with exception of TB) after 6 months of effective treatment [15].
  Immunological failure: children younger than 5 yearsexhibit persistent CD4 levels below 200 cells/mm^3^, and those older than 5 years exhibit persistent CD4 levels below 100 cells/mm^3^ [15].
  Virological failure is defined as viral load above 1000 copies/mL based on two consecutive viral load measurements in 3 months with adherence support following the first viral load test. An individual must be taking ART for at least 6 months before it can be determined that the regimen has failed [15].
  First-line antiretroviral treatment failure: in this study, treatment failure was considered when, due to virological failure, first-line regimen is changed to second-line regimen.

### 2.6. Data Collection Technique and Procedure

Three health facilities were selected based on the duration of the ART service. Data extraction tools were adapted from the revised 2017 Ethiopian Federal Ministry of Health HIV Care/ART Follow-Up Form, which was used in the ART clinic and by using different peer-reviewed published literature [6–8, 16–20]. Secondary data from three public health facilities of Shashamene town were used to retrieve data from the initial date of ART up to the end of the follow-up (from January 1, 2011, to December 30, 2015, and followed up until February 30, 2018). All HIV-positive children on care and support follow-up who had started ART at public health facilities of Shashamene town and fulfilled the inclusion criteria were included in the study.

### 2.7. Data Quality Assurance

The quality of the data was assured by caring out a careful design of the checklist. Training was given for data collectors and supervisors and a pretest was conducted before data collection. Finally, five percent of the completed data was selected randomly and cross-checked with medical cards of the patients to check for consistency at the end of each day. The collected data were checked for its completeness manually. Data coding and entry was done using EpiData version 3.1.

### 2.8. Data Analysis Procedure

The data were exported from EpiData to SPSS version 20 for data analysis. Data organizing was done to transform the data into a format suitable for further analysis. Finally, two standard statistical methods were employed: a nonparametric method called the Kaplan–Meier method and the associated log-rank test, and a semiparametric method known as the Cox proportional hazards regression model. The Kaplan–Meier method was used to estimate the survival probability after the initiation of ART to first-line treatment failure, and a log-rank test was used to compare survival curves of children between different categories of predictor variables at *p* value (<0.05).

The Cox proportional hazard regression model was used to determine the predictors of time to first-line treatment failure. In the bivariable model, covariables with *p* values (<0.25) were selected to be included in the multivariable model. The interaction effect was assessed for possible covariables at *p* value of 0.05, and the model fitness was checked using log minus log-survival curve for predictor variables. The final model was interpreted using the adjusted hazard ratio (AHR) with 95% confidence interval and *p* value <0.05 to measure the predictors of the first-line treatment failure.

### 2.9. Ethical Consideration

Ethical clearance was obtained from the Institutional Review Board (IRB) of Institute of Health, Jimma University. In addition, permission letter was sought from West Arsi Zonal Health Office and each health facility administrative. The retrieved data were kept strictly confidential and the names of children or their parents were not included.

## 3. Results

### 3.1. Sociodemographic Characteristics

A total of 484 medical cards of children living with HIV/AIDS and who started ART drugs were reviewed, of which 70 (14.5%) were excluded following the exclusion criteria. Finally, a total of 414 children were included in the analysis. Half (212 (51.2%)) of the children were male. Majority of them 268 (64.7%) were above 5 years at the initiation of ART. The mean age of children at the initiation of ART was 6.6 years with (SD ± 3.8) (Table 1).

#### 3.1.1. Baseline Clinical Status of the Study Subjects

Among 414 HIV-positive children enrolled on ART, 383 (92.5%) and 202 (48.8%) of them received cotrimoxazole and isoniazide prophylaxis initiation, respectively. Of the study participants, 85 (20.5%) were anemic and 128 (30.9%) had CD4 count below the threshold level during ART initiation (Table 2).

#### 3.1.2. Outcome and Survival Status of Children

From a total of children initiated HAART during the study period, 27 (6.5%) of them had virological failure, 17 (4.1%) had immunological failure, 20 (4.8%) had clinical failure, 37 (8.9%) died, 47 (11.4%) were lost to follow-up, 44 (10.6%) were transferred out, and 222 (55.6%) were active. The median follow-up time was 38 months with interquartile range (IQR) of 24 to 52 months, and the median survival time to virological failure was 30 months with IQR of 24.4 to 44.2.

**Figure 1:**
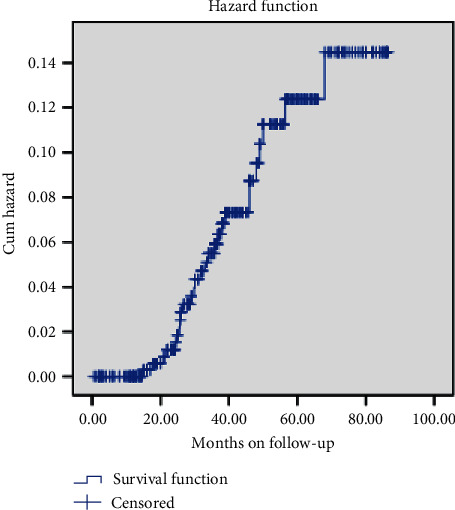
Cumulative probability of first-line ART failure among patients in Shashamene town health facilities.

The cumulative probability of survival status of children after 1^st^, 2^nd^, 3^rd^, and 4^th^ years was 81%, 41%, 19%, and 4%, respectively (Figure 1). This indicates that as the child received ART for a long period of time, the probability of treatment failure increases. The incidence density of treatment failure was 16 children per 10,000 person months with a total of 16,583 person months of follow-up.

#### 3.1.3. Comparison of Survival Functions

**Figure 2:**
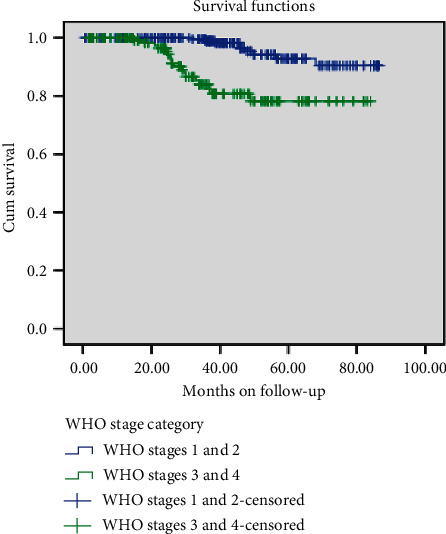
Survival curves for the cohort of children on ART according to their WHO clinical stage.

**Figure 3:**
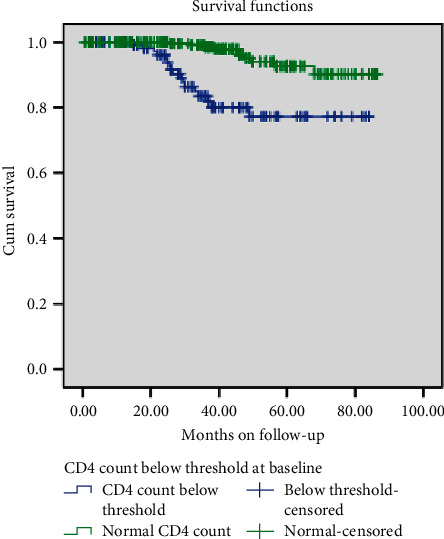
Survival curves for the cohort of children on ART according to their CD4 count.

Children in WHO clinical stages one and two survived significantly longer than those in clinical stages three and four after HAAR initiation before treatment failure (log-rank, *p* value = 0.001). Children with CD4 count above the threshold level at baseline survived more before treatment failure compared to their counterparts. This difference was also statistically significant (log-rank, *p* value = 0.003) (Figures 2 and 3).

#### 3.1.4. Sociodemographic Predictors of Time to Treatment Failure among Children Started ART

From sociodemographic factors during bivariable Cox regression, only sex being male was nominated for multivariable analysis (Table 3).

### 3.2. Bivariable Cox Regression Analysis for Clinical Predictors

From clinical factors during bivariable Cox regression, having anemia during the start of ART, TB infection during the follow-up, baseline advanced WHO clinical stages (stages 3 and 4), baseline CD4 count below threshold level, and usage of nevirapine at baseline were nominated for multivariable Cox regression analysis (Table 4).

#### 3.2.1. The Overall Predictors of Time to First-Line Treatment Failures among HIV-Infected Children on ART

From the multivariable Cox regression analysis, children in WHO stages 3 and 4 during the initiation of ART were 5.7 times at higher risk of treatment failure (AHR = 5.69 (95% CI: 2.07–15.66) compared to children in WHO stages 1 and 2; and children treated with NVP-based NNRT initially were 2.7 times at higher risk of treatment failure (AHR = 2.72 (1.13–6.54)) compared to children treated with non-NVP (Table 5).

## 4. Discussion

The study has provided pertinent information about predictors with time to treatment failure among children living with HIV enrolled on ART which can support activities being implemented to prevent the occurrence of early first-line treatment failure for planners and decision makers. From a total of children initiated HAART during the study period, 27 (6.5%) of them had virological failure with the median survival time of 30 months with an IQR of 24.42 to 44.25. This time was similar to that reported in the study done in South London; the median duration of antiretroviral therapy at treatment failure was 30 months (range: 13 months–5 years) [21] but it was high as compared to the study done in Uganda and Mozambique [6]. This difference could be due to the difference in follow-up period and the study settings.

The incidence density of the treatment failure in this study was 16 children per 10,000 person months with a total of 16,583 person months of follow-up. This result was low as compared to the study done in Amhara Regional State, which was 22.1 per 10,000 person months [22]. The reason for the lowest incidence density could be related to the difference in the study period/setting and this finding focused only on virological failure.

Children in the advanced WHO clinical stage (stages 3 and 4) during the initiation of ART were 5.7 times at higher risk of treatment failure than their counterparts at WHO stages 1 and 2, which is consistent with the study done in Mozambique and Uganda; children in WHO stages 3 and 4 were significantly more likely to experience treatment failure [6]. This could be due to the fact that children in advanced WHO clinical stages are more likely to have advanced immune suppression and higher rate of comorbidities so that the risk of ART failure could be higher.

Children treated with NVP-based NNRT initially were 2.7 times at higher risk of treatment failure compared to their counterparts. This result was in line with the study done in South Africa, where the use of nevirapine-based regime in the initial stage increases the risk for early treatment failure [23]. The study done in Uganda and Tanzania on predictors of virological failure also supports this finding [24, 25]. This could be due to low dose of NVP drug transmission to infants through placenta or breast feeding during PMTCT.

The strength and limitations of this study are as follows:

  Strength: the study uses a strong design and a longer follow-up duration to estimate the survival time and independent predictors of first-line ART failure
  Limitation: restriction of incomplete data may underestimate or overestimate this result; there are limited data on key possible predictors of VF such as tuberculosis coinfection and PMTCT. Missing data on variables may limit the range of variables and the number of children that could be included in multivariable models.

## 5. Conclusion and Recommendation

The median survival time of first-line antiretroviral treatment failure was moderate in the study area as compared to other studies. The finding also demonstrated that children treated with NVP-based NNRT at the initial and advanced WHO clinical stages at baseline were at higher risk of treatment failure. Therefore, Ministry of Health, stakeholders, and healthcare providers should give attention to ensure early diagnosis and enrollment into ART. Health workers need to give more emphasis during clinical care for patients taking NVP-based NNRT treatment and who are in advanced WHO clinical stages. The finding of increased rates of virological failure among children receiving nevirapine suggests that more work should be done by the Ministry of Health to make efavirenz a cost-effective option for pediatric antiretroviral treatment programs in the country.

## Figures and Tables

**Table 1:** Sociodemographic characteristics of children who started ART at public health facilities between January 1, 2011, to December 30, 2015, in Shashamene town, Oromia Region, Ethiopia, 2019.

Variables	Category	No.	(%)
Sex	Female	202	48.8
	Male	212	51.2

Age	<1 year	27	6.5
	1–5 years	119	28.7
	≥5 years	268	64.7

Child's caregiver	Parents	335	80.9
	Sibling	64	15.5
	Orphan	13	3.1

Nutritional history	Exclusive breastfeeding	147	35.5
	No exclusive breastfeeding	261	63
	Appropriate for age	312	75.9

Developmental milestone	Delayed	76	18.5
	Regressed	23	5.6

Immunization status	Appropriate for age	218	52.7
	Not appropriate for age	140	33.8
	Not immunized	54	13

**Table 2:** Baseline clinical status among children who started ART at public health facilities between January 1, 2011, and December 30, 2015, in Shashamene town, Oromia Region, Ethiopia, 2019.

Variables	Category	No.	(%)
Cotrimoxazole prophylaxis initiation	Yes	383	92.5
	No	31	7.5

Isoniazide prophylaxis initiation	Yes	203	49
	No	211	51

Baseline Hgb status	Anemic	85	20.5
	Normal	319	77.1

TB smear test result during follow-up	Positive	90	21.7
	Negative	303	73.2

Baseline CD4 count	Below threshold level	128	30.9
	Normal	286	69.1

Baseline WHO stage	Stages 1 and 2	271	65.5
	Stages 3 and 4	143	34.5

Baseline nutritional status	Normal	217	52.4
	Undernutrition	197	47.7

Exposure status for PMTCT	Exposed	132	31.9
	Not exposed	197	47.6

Regime	Nonnevirapine	172	41.5
	Nevirapine	240	58

Adherence	Good	251	60.6
	Fair	82	19.8
	Poor	80	19.3

**Table 3:** Bivariable Cox regression analysis for sociodemographic determinants of survival among children started ART at public health facilities between January 1, 2011, and December 30, 2015, at Shashamene town, Oromia Region, Ethiopia.

Variables	Category	No. (%)	CHR (95% CI)	*p* value
Sex	Female	202 (48.8)	1	
	Male	212 (51.2)	1.72 (0.727–4.05)	0.21

Age	<1 year	27 (6.5)	0.305 (0.39–2.421)	0.26
	1–5 years	119 (28.7)	1.311 (0.548–3.13)	0.54
	≥5 years	268 (64.7)	1	

Child's caregiver	Parents	335 (80.9)	1	
	Sibling	64 (15.5)	1.35 (0.49–3.68)	0.55
	Orphan	13 (3.1)		

Nutritional history	Exclusive breastfeeding	147 (35.5)	1	
	No exclusive breastfeeding	261 (63)	0.93 (0.37–2.75)	0.9
	Appropriate for age	312 (75.9)	1	

Developmental milestone	Delayed	76 (18.5)	1.24 (0.50–3.06)	0.64
	Regressed	23 (5.6)	2 (0.56–7.14)	0.28

Immunization status	Appropriate for age	218 (52.7)	1	
	Not appropriate for age	140 (33.8)	1.0 (0.39–2.56)	0.99
	Not immunized	54 (13)	0.78 (0.28–2.21)	0.65

**Table 4:** Bivariable Cox regression analysis for clinical determinants of survival among children who started ART at public health facilities between January 1, 2011, to December 30, 2015, at Shashamene town, Oromia region, Ethiopia.

Variables	Category	No. (%)	CHR (95% CI)	*p* value
Cotrimoxazole prophylaxis initiation	Yes	383 (92.5)	1	
	No	31 (7.5)	0.96 (0.39–7.27)	0.97

Isoniazide prophylaxis initiation	Yes	202 (48.8)	1	
	No	211 (51)	1.53 (.59–3.92)	0.37

Baseline Hgb status	Anemia	85 (20.5)	3.87 (1.42–10.57)	0.01
	Normal	319 (77.1)	1	

TB result	Positive	90 (21.7)	2.18 (0.89–5.36)	0.08
	Negative	303 (73.2)	1	

Baseline CD4 count	Below threshold level	128 (30.9)	4.37 (1.37–11.14)	0.002
	Normal	286 (69.1)	1	

Baseline WHO stage	Nonadvanced stage (stages 1 and 2	271 (65.5)	1	
	Advanced stage (stages 3 and 4)	143 (34.5)	5.35 (2.0–14.30)	0.001

Baseline nutritional status	Z score > −2	143 (34.5)	1	
	Z score ≤ −2	24 (5.8)	1.43 (.28–7.84)	0.66
	BMI < 16 kg/m^2^	271 (65.5)	1.59 (0.64–4.07)	0.33
	BMI ≥ 16 kg/m^2^	143 (34.5)	1	

Exposure status for PMTCT	Exposed	132 (31.9)	1.23 (0.48–3.15)	0.66
	Not exposed	197 (47.6)	1	

Regime	Nonnevirapine	172 (41.5)	1	
	Nevirapine	240 (58.0)	2.43 (1.04–5.67)	0.04

Adherence	Good	251 (60.6)	1	
	Fair	82 (19.8)	0.646 (0.13–3.29)	0.6
	Poor	81 (19.6)	1.349 (0.39–4.61)	0.63

**Table 5:** The overall predictors of time to first-line treatment failure among children started ART.

Variables	Category	AHR (95% CI)	*p* value
Sex	Male	1	
	Female	1.07 (0.37–3.02)	0.9

Hgb level	Anemia	2.28 (0.77–6.78)	0.14
	No anemia	1	

TB result	Positive	1.30 (0.45–3.71)	0.63
	Negative		

Baseline CD4 count	Below threshold level	1.13 (0.29–9.96)	0.91
	Normal		

Baseline WHO stage	Stages 1 and 2	1	
	Stages 3 and 4	5.69 (2.07–15.66)	0.001

Treatment regime	Nonnevirapine	1	
	Nevirapine	2.72 (1.13–6.54)	0.02

## Data Availability

The raw datasets used and/analyzed during the current study are available from the corresponding author on reasonable request.

## Abbreviations

AHR: Adjusted hazard ratio
ART: Antiretroviral therapy
ARTF: Antiretroviral therapy failure
BMI: Body mass index
CHR: Crude hazard ratio
cART: Combination antiretroviral therapy
EPHIC: Ethiopian Pediatric HIV Cohort
HAART: Highly active antiretroviral therapy
HIV/AIDS: Human immunodeficiency virus/acquired immunodeficiency syndrome
IQR: Interquartile range
LMIC: Low-middle-income countries
NNRT: Nonnucleotide reverse transcriptase
NVP: Nevirapine
OI: Opportunistic illness
PLWH: People living with HIV
PMTCT: Prevention of mother-to-child transmission
WHO: World Health Organization.
