# Corrigendum to “Time to First-Line Antiretroviral Treatment Failure and Its Predictors Among HIV-Positive Children in Shashemene Town Health Facilities, Oromia Region, Ethiopia, 2019”

**DOI:** 10.1155/tswj/9820382

**Published:** 2025-06-27

E. Zenebe, A. Washo, and A. A. Gesese, “Time to First-Line Antiretroviral Treatment Failure and Its Predictors Among HIV-Positive Children in Shashemene Town Health Facilities, Oromia Region, Ethiopia, 2019”, *The Scientific World Journal*, vol. 2021 (2021). https://doi.org/10.1155/2021/8868479.

In the article titled “Time to First-Line Antiretroviral Treatment Failure and Its Predictors Among HIV-Positive Children in Shashemene Town Health Facilities, Oromia Region, Ethiopia, 2019”, Dr. Solomon Berhanu and Dr. Henok Asefa were missing from the author list.

Dr. Solomon Berhanu and Dr. Henok Asefa contributed to the conception and design of the study in addition to collecting, analyzing, and interpreting the data and manuscript preparation.

The corrected author list should read as follows:

[“Endale Zenebe^1^, Assefa Washo^2^, Abreham Addis Gesese^1^, Solomon Berhanu^1^, and Henok Asefa^1^

^1^Jimma University, College of Public Health and Medical Science, Department of Epidemiology, Jimma, Ethiopia

^2^Hawassa College of Health Science, Department of Public Health, Hawassa, Ethiopia”]

All authors agree with the abovementioned changes.

We apologize for this error.

